# Glycyrrhizic acid improved lipoprotein lipase expression, insulin sensitivity, serum lipid and lipid deposition in high-fat diet-induced obese rats

**DOI:** 10.1186/1476-511X-9-81

**Published:** 2010-07-29

**Authors:** Chia Hui Apphia Eu, Wai Yen Alfred Lim, So Ha Ton, Khalid bin Abdul Kadir

**Affiliations:** 1 School of Science, Monash University Sunway Campus, Jalan Lagoon Selatan, Bandar Sunway 46150, Selangor Darul Ehsan, Malaysia; 2 School of Medicine and Health Sciences, Monash University Sunway Campus, Jalan Lagoon Selatan, Bandar Sunway 46150, Selangor Darul Ehsan, Malaysia

## Abstract

**Background:**

The metabolic syndrome, known also as the insulin resistance syndrome, refers to the clustering of several risk factors for atherosclerotic cardiovascular disease. Dyslipidaemia is a hallmark of the syndrome and is associated with a whole body reduction in the activity of lipoprotein lipase (LPL), an enzyme under the regulation of the class of nuclear receptors known as peroxisome proliferator-activated receptor (PPAR). Glycyrrhizic acid (GA), a triterpenoid saponin, is the primary bioactive constituent of the roots of the shrub *Glycyrrhiza glabra*. Studies have indicated that triterpenoids could act as PPAR agonists and GA is therefore postulated to restore LPL expression in the insulin resistant state.

**Results:**

Oral administration of 100 mg/kg of GA to high-fat diet-induced obese rats for 28 days led to significant reduction in blood glucose concentration and improvement in insulin sensitivity as indicated by the homeostasis model assessment of insulin resistance (HOMA-IR) (p < 0.05). LPL expression was up-regulated in the kidney, heart, quadriceps femoris, abdominal muscle and the visceral and subcutaneous adipose tissues but down-regulated in the liver - a condition in reverse to that seen in high-fat diet-induced obese rats without GA. With regard to lipid metabolism, GA administration led to significant hypotriglyceridemic and HDL-raising effects (p < 0.05), with a consistent reduction in serum free fatty acid, total cholesterol and LDL cholesterol and significant decrease in tissue lipid deposition across all studied tissue (p < 0.01).

**Conclusion:**

In conclusion, GA may be a potential compound in improving dyslipidaemia by selectively inducing LPL expression in non-hepatic tissues. Such up-regulation was accompanied by a GA-mediated improvement in insulin sensitivity, which may be associated with a decrease in tissue lipid deposition. The HDL-raising effect of GA suggests the antiatherosclerotic properties of GA.

## Background

The metabolic syndrome (MetS), known also as the insulin resistance syndrome and syndrome X, refers to the clustering of several risk factors for atherosclerotic cardiovascular disease. Insulin resistance (IR) is recognized as the potential underlying etiology of the various metabolic abnormalities that comprises the syndrome, of which includes abdominal obesity, glucose intolerance, atherogenic dyslipidaemia and hypertension. Individuals with the syndrome are at a 2- and 5-fold increased risk for the development of cardiovascular diseases and diabetes mellitus respectively [1-3].

Various studies have shown that with the development of IR, the production and activity of the enzyme lipoprotein lipase (LPL) is reduced [4,5]. LPL acts to hydrolyze the core triacylglycerol (TAG) of circulating TAG-rich lipoproteins to regulate the entry of fatty acids into the underlying tissues [5,6]. Reduced LPL activity has been shown to lead to an inhibition in the lipolytic rate of the chylomicrons and the very-low-density lipoproteins (VLDL), triggering the development of hypertriglyceridaemia and the subsequent dyslipidaemia seen in the MetS [7,8].

Stimulation of LPL activity by either the use of transgenic overexpression or the administration of LPL-raising drugs has been shown to ameliorate the observed dyslipidaemia. Systemic overexpression of the human LPL transgene in Watanabe heritable hyperlipidemic (WHHL) rabbits which mimic the features of MetS in humans, for example, led to a significant decrease in plasma TAG levels and corrected the hypercholesterolemia in these animal models [9]. In addition, targeted over-expression of LPL in skeletal muscle of transgenic mice lowered both plasma TAG and FFA levels even upon high-fat feeding [10].

Currently available classes of LPL-raising drugs include the fibrates, thiazolidinediones (TZDs) and the NO-1886 (ibrolipim). Both the fibrates and TZD are peroxisome proliferator-activated receptor (PPAR) agonists that act on the isoforms PPAR-α [7] and PPAR-γ [11] respectively to induce an increase in LPL activity. These PPAR nuclear receptors then bind to the peroxisome proliferator response element (PPRE) present in the promoter region of the LPL gene [12]. Despite the effectiveness of fibrates however, patients on long-term fibrate therapy suffer from an increased incidence of cholesterol gallstone [13]. The TZDs on the other hand is associated with fluid retention and plasma volume expansion which lead to peripheral edema. This increases the incidence of heart failure in some patients, with a frequency of 2.5 times greater in patients on combination therapy with insulin [14]. Lastly, NO-1886 has been reported to inhibit basal and adrenocorticotrophic hormone (ACTH)-induced release of steroid hormones in rat, dog, monkey and human adrenocortical cells, resulting in hypertrophy of the adrenals [15]. With such drawbacks, the development of an alternative LPL-promoting agent is therefore needed.

Glycyrrhizic acid (GA), a triterpenoid saponin, is the primary bioactive constituent of the roots of the shrub *Glycyrrhiza glabra*. Interest in GA arose following discoveries that excess tissue glucocorticoid action, amplified by the enzyme 11β-hydroxysteroid dehydrogenase type 1 (11β-HSD1), could promote the symptoms of the MetS. GA and its active metabolite glycyrrhetic acid act as potent inhibitors of 11β-HSD1 [16]. More importantly however, is that triterpenoids had been discovered to act as PPAR agonists [17,18] and this may suggest that GA could also potentially act to activate the PPAR class of nuclear receptors and may therefore be proposed to be a candidate for raising LPL. In this study, the expression of LPL, insulin sensitivity and lipid parameters are compared between high-fat diet-induced obese rats treated and non-treated with GA.

## Results

### GA treatment restored LPL expression in obese-induced rats

**Figure 1:**
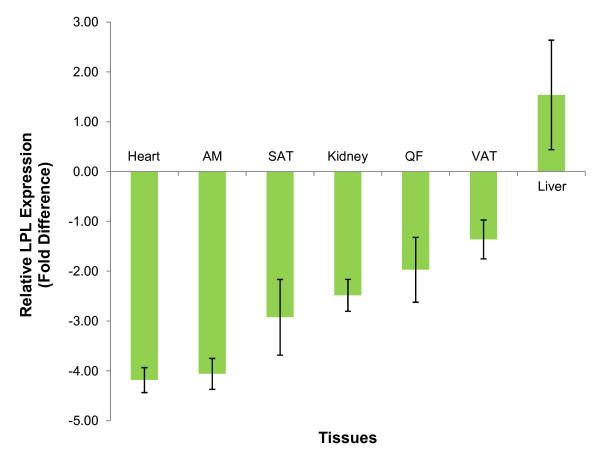
**Relative LPL expression of Group B compared to A**. Fold difference of LPL expression in tissues studied, using β-actin (BAC) as the endogenous reference, tissues of rats from group A as the calibrator and tissues of rats from group B as the target. LPL expression was down-regulated in all tissues except the liver. [Group A: rats fed on normal diet without GA; Group B: rats fed on high-fat diet without GA].

**Figure 2:**
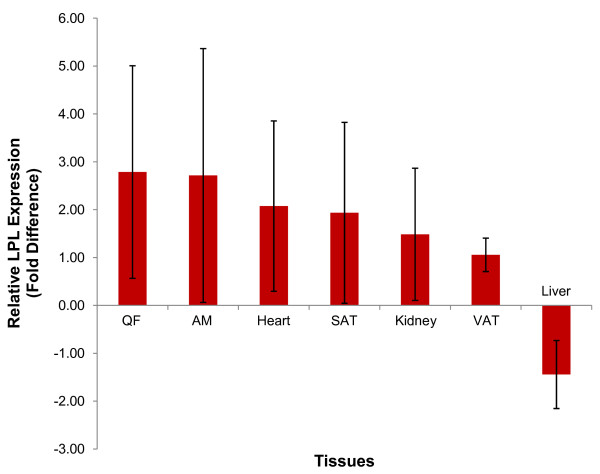
**Relative LPL expression of Group C compared to B**. Fold difference of LPL expression in tissues studied, using BAC as the endogenous reference, tissues of rats from group B as the calibrator and tissues of rats from group C as the target. LPL expression was up-regulated in all tissues except the liver. [Group B: rats fed on high-fat diet without GA; Group C: rats fed on high-fat diet and given 100 mg/kg of GA].

In comparing between rats on normal diet (group A) and rats on high-fat diet (group B), LPL expression was downregulated in all tissues in the latter except the liver (Figure 1). The heart showed the highest decrease with a fold difference of -4.184 ± 0.25, followed by the abdominal muscle (AM) (- 4.059 ± 0.31), kidney (-2.483 ± 0.32 fold), subcutaneous adipose tissue (SAT) (-2.924 ± 0.39 fold), quadriceps femoris (QF) (-1.970 ± 0.65) and visceral adipose tissue (VAT) (-1.361 ± 0.76). No significant differences were seen in any of the tissues when comparing the two groups (p > 0.05). In the liver, LPL was up-regulated by 1.539 ± 1.10 fold (p > 0.05). When comparing rats from group B with group C (rats on high-fat diet and given 100 mg/kg of GA), the reverse was observed where LPL expression was up-regulated in all tissues in the latter group (Figure 2) except the liver. The highest up-regulation was observed in the QF (fold difference = 2.786 ± 2.22) followed by the AM (2.715 ± 2.65), heart (2.076 ± 1.78), SAT (1.935 ± 1.89), kidney (1.486 ± 1.38) and VAT (1.058 ± 0.35). These increases were not significant (p > 0.05). LPL expression in the liver was down-regulated by a fold of -1.443 ± 0.71 and this was not significant (p > 0.05).

### GA treatment improved insulin sensitivity in obese-induced rats

**Figure 3:**
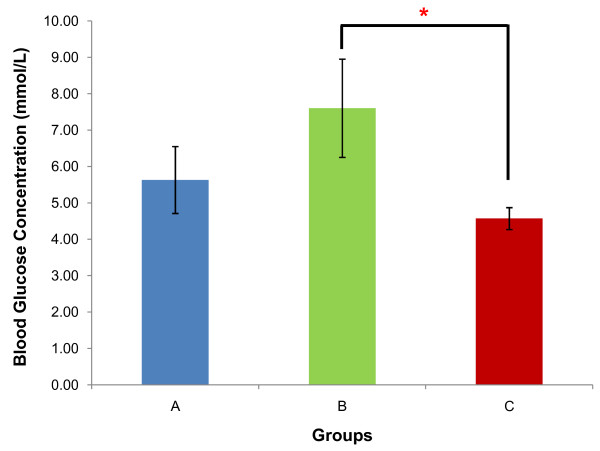
**Comparison of blood glucose**. Mean concentration of blood glucose (mmol/L) of rats from groups A, B and C. * indicates p < 0.05 when compared between groups. [Group A: rats fed on normal diet without GA; Group B: rats fed on high-fat diet without GA; Group C: rats fed on high-fat diet and given 100 mg/kg of GA].

**Figure 4:**
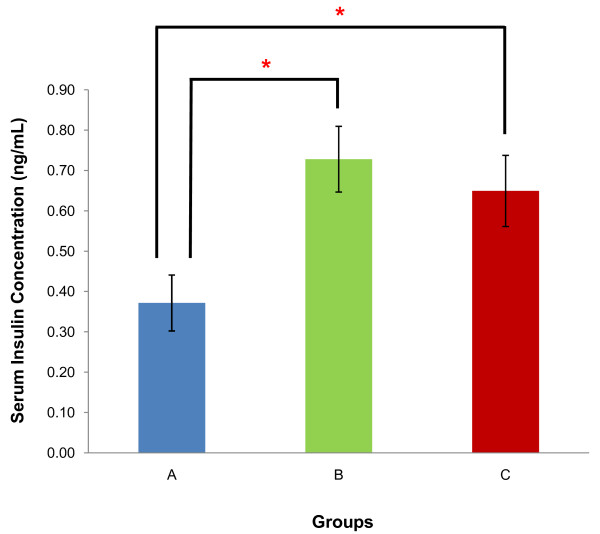
**Comparison of serum insulin**. Mean concentration of serum insulin (ng/mL) of rats from groups A, B and C. * indicates p < 0.05 when compared between groups. [Group A: rats fed on normal diet without GA; Group B: rats fed on high-fat diet without GA; Group C: rats fed on high-fat diet and given 100 mg/kg of GA].

**Figure 5:**
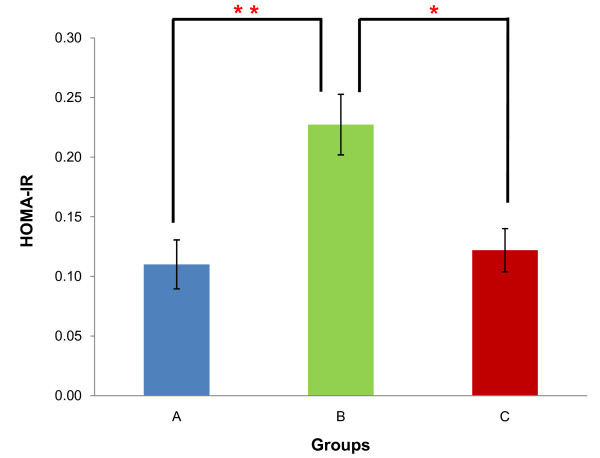
**Comparison of HOMA-IR**. Mean HOMA-IR of rats from groups A, B and C. ** indicates p < 0.01 and * indicates p < 0.05 when compared between groups. [Group A: rats fed on normal diet without GA; Group B: rats fed on high-fat diet without GA; Group C: rats fed on high-fat diet and given 100 mg/kg of GA].

Mean blood glucose concentrations of rats from groups A, B and C were 5.63 ± 0.92 mmol/L, 7.60 ± 1.35 mmol/L and 4.57 ± 0.30 mmol/L respectively (Figure 3). Rats from group C presented a significant decrease compared to group B (p < 0.05) but non-significant decrease compared to group A (p > 0.05). Mean serum insulin concentrations of rats from groups A, B and C were 0.37 ± 0.07 ng/mL, 0.73 ± 0.08 ng/mL and 0.65 ± 0.09 ng/mL respectively (Figure 4). This represented a significant increase when comparing between group A with groups B (p < 0.05) and C (p < 0.05). However no significant difference was seen between groups B and C (p > 0.05). Analysis of the HOMA-IR showed that rats from group B (0.23 ± 0.03) had the highest HOMA-IR index compared to groups A (0.11 ± 0.02) and C (0.12 ± 0.02) with a significant increase in groups B relative to A (p < 0.01) but a non-significant increase in groups C relative to A (p > 0.05). The HOMA-IR index decrease in group C was significant compared to group B (p < 0.05) (Figure 5).

### GA treatment led to a positive shift in serum lipid in obese-induced rats

**Figure 6:**
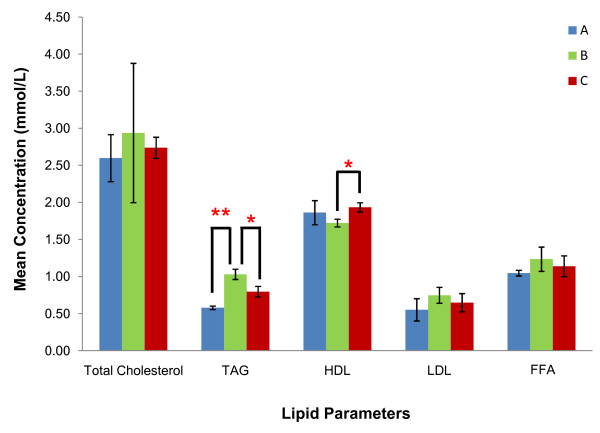
**Comparison of serum lipid**. Mean concentration of total cholesterol, TAG, HDL-cholesterol, LDL-cholesterol and serum FFA in rats from groups A, B and C. ** indicates p < 0.01 and * indicates p < 0.05 when compared between groups. [Group A: rats fed on normal diet without GA; Group B: rats fed on high-fat diet without GA; Group C: rats fed on high-fat diet and given 100 mg/kg of GA].

Overall, rats from group C had improved lipid profile with a 6.80% decrease in total cholesterol, 22.33% decrease in TAG, 13.33% decrease in LDL, 12.21% increase in HDL and 7.32% decrease in serum FFA (Figure 6). Comparison of lipid profiles of rats from groups A, B and C showed that mean total cholesterol was the highest in rats from group B (2.94 ± 0.94 mmol/L) compared to groups A (2.60 ± 0.32 mmol/L) and C (2.74 ± 0.14 mmol/L). No significant difference was observed between all groups. TAG concentrations in groups A, B and C were 0.58 ± 0.02, 1.03 ± 0.07 and 0.80 ± 0.07 mmol/L respectively. A significant increase (77.59%) was observed in group B compared to A (p < 0.01) while a significant decrease (22.33%) was seen in group C compared to B (p < 0.05). HDL concentrations in groups A, B and C were 1.86 ± 0.16, 1.72 ± 0.05 and 1.93 ± 0.06 mmol/L respectively and this represented a significant increase (12.21%) in group C compared to B (p < 0.05). LDL concentrations showed no significant difference between all three groups which has a level of 0.55 ± 0.15 (group A), 0.75 ± 0.11 (group B) and 0.65 ± 0.12 (group C) mmol/L respectively. Lastly, FFA levels were 1.05 ± 0.04, 1.23 ± 0.16 and 1.14 ± 0.14 mmol/L respectively for groups A, B and C with no significant difference between any groups.

### GA treatment reduced tissue lipid deposition in obese-induced rats

**Figure 7:**
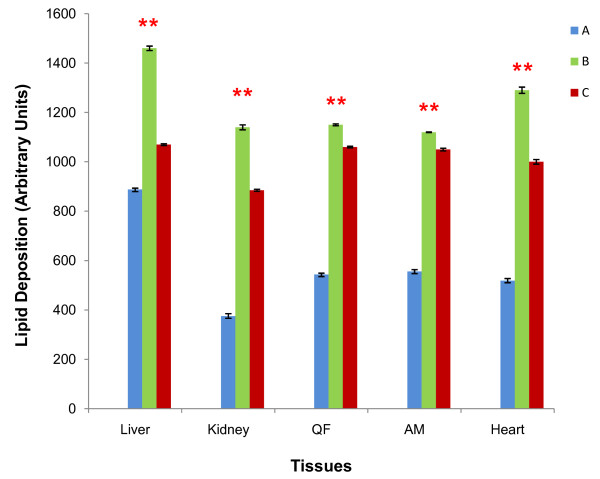
**Comparison of tissue lipid deposition**. Lipid deposition in tissues studied, measured in arbitrary units (AU). Sections of VAT and SAT were used as positive controls. ** indicates significant increase in lipid deposition when comparing between Group A with each of Groups B and C, and a significant decrease in lipid deposition in Group C compared to B (p < 0.01). [Group A: rats fed on normal diet without GA; Group B: rats fed on high-fat diet without GA; Group C: rats fed on high-fat diet and given 100 mg/kg of GA].

Tissue lipid deposition was significantly increased in all tissues of groups B and C compared to A, and was significantly reduced in all tissues of group C compared to B (p < 0.01 in all cases) (Figure 7). Mean lipid deposition in all the five lobes of the liver from groups A, B and C were 8.88 × 10^2 ^± 5.45 AU, 1.46 × 10^3 ^± 9.06 AU and 1.07 × 10^3 ^± 3.64 AU respectively. This represented an increase of 39.18% and 17.00% respectively when comparing groups B and C with A and a decrease of 26.71% in group C compared to B. Mean lipid deposition in both kidneys from groups A, B and C were 3.75 × 10^2 ^± 10.39 AU, 1.41 × 10^3 ^± 10.04 AU and 8.85 × 10^2 ^± 4.12 AU respectively, indicating a 67.11% and 57.63% increase in groups B and C relative to A and a 22.37% decrease in group C relative to B. For the three muscle groups studied, QF lipid deposition showed a 52.70% and 48.68% increase in groups B and C relative to A and 7.83% decrease in group C relative to B (group A, 44 × 10^2 ^± 5.02 AU; B, 1.15 × 10^3 ^± 3.74 AU; C, 1.06 × 10^3 ^± 3.00 AU), AM lipid deposition charted a 50.36% and 47.05% increase in groups B and C relative to A and an accompanying 6.25% decrease in group C relative to B (group A, 5.56 × 10^2 ^± 7.53 AU; B,1.12 × 10^3 ^± 1.44 AU; C, 1.05 × 10^3 ^± 5.47 AU) and heart lipid deposition showed an increase of 59.77% and 48.1% in groups B and C relative to A and a decrease of 22.48% in group C relative to B.

## Discussion

Numerous studies have revealed that high-fat diets promote hyperglycemia and whole-body IR. It is generally accepted that high-fat diets can be used to generate a valid rodent model for the MetS with IR [19]. Overfeeding of animals with high-fat diet of more than 50% of calories as fat for approximately five weeks is sufficient to initiate moderate obesity and often results in IR [20,21]. In the present study, rats were fed a high-fat diet with 60% of calories from vegetable shortening for four weeks. Rats fed a high-fat diet consumed significantly more calories on a per day basis compared to the controls which were only fed standard rat chow.

In the insulin resistant state, the decrease in insulin-mediated suppression of lipolysis in adipocytes promotes the release of fatty acids which inhibits LPL activity [22]. When supply of fatty acids exceeds tissue demand, fatty acids would probably bind to LPL and displace it from its binding sites, thereby rendering them non-functional [23]. LPL expression of rats fed on high-fat diet without GA (group B) was down-regulated in all non-hepatic tissues compared to the controls (group A). The down-regulation of LPL in the adipose tissues, muscles and kidney under study may be due to inflammatory mediators such as tumour necrosis factor-alpha (TNF-α) which are found to be elevated in obesity and insulin resistant states [24]. Its inhibition of LPL gene transcription is suggested to be mediated in part by blocking the nuclear-factor-Y/CCAAT interactions with LPL promoter [6,25]. However, the increase in TNF-α level renders an opposing effect in the liver. According to Wang and Eckel [26], LPL is not normally expressed in the adult liver of animals but can be expressed under specific physiological and pathological conditions. A single dose of TNF-α can cause a significant increase in LPL mRNA levels in the liver. However, the detailed mechanism of such induction is not well understood. The increase in hepatic LPL activity and its concomitant decrease in the non-hepatic tissues could have resulted in greater partitioning of plasma TAG to the liver and increased hepatic uptake of FFAs. This would lead to an increase in the secretion of VLDL-TAG and apolipoprotein B100 (apoB100) from the liver [27], and hence may account for the observed hypertriglyceridaemia in group B compared to group A.

In rats on high-fat diet given GA (group C), LPL expression was up-regulated in all non-hepatic tissues, a condition that opposes that seen in group B. It was postulated that GA results in the up-regulation of LPL expression via the activation of PPAR class nuclear receptors since the LPL gene was found downstream of the transcriptionally active PPRE [5]. Of the tissues in which LPL expression was up-regulated, the highest up-regulation took place mainly in the muscles (AM, QF and heart) compared to the SAT and VAT (Figure 2). This suggest that GA may exhibit a higher potency in activating PPARα than PPARγ; PPARα is highly expressed in the heart, muscles, liver and kidney in which it has a crucial role in controlling fatty acid oxidation [28], while PPARγ is highly expressed in the adipocytes where it triggers adipocyte differentiation and lipogenesis [6]. The activation of PPARα may lead to a direct up-regulation of LPL expression and also down-regulate apo- CIII, an inhibitor of LPL [29] that is up-regulated in the insulin resistant state [8]. The downregulation of LPL expression in the liver might be due to the reduction in macrophage-derived TNF-α in the adipose tissue. According to Jeong and Yoon [30], PPARα activation in adipose tissue decrease mRNA levels of TNF-α which eventually inhibit adipocyte hypertrophy in obese animals. Hence, GA is postulated to activate PPARα in the adipose tissues to decreases TNF-α production and subsequently down-regulate LPL in the liver. The end effect of these is that GA thus promotes partitioning of lipids away from the liver into the oxidative tissues.

Improvement in lipid profile following GA treatment in obese-induced rat was similar to that previously reported by Lim *et al. *[31] in lean rats, but with a more prominent hypotriglyceridemic and HDL-raising effect. The hypertriglyceridaemia observed in patients with the MetS and T2DM originates from (i) lipolysis of TAG store from adipose tissue that causes elevated FFA flux to the liver and hence, increased hepatic TAG synthesis and (ii) inhibition of lipolysis of chylomicrons and VLDL due to decreased LPL levels [8]. Our present study indicated that GA administration in obese rats could curb such development by its selective induction of LPL expression in the non-hepatic tissues to promote catabolism of circulating TAG-rich lipoproteins and prevent further uptake of FFA into the liver by down-regulating hepatic LPL expression. More importantly, GA induced a significant increase in HDL levels in the obese rats. Elevating HDL-cholesterol may serve as a more attractive treatment alternative instead of lowering LDL cholesterol as dyslipidaemia is often characterized by a normal range of serum LDL-cholesterol, but with a predominance of the more atherogenic small, dense LDL rather than the less atherogenic large, buoyant LDL particles [7]. The atheroprotective effect of HDL is exerted through its ability to counteract LDL oxidation, the major initiating event that prompts the development of atherosclerosis. The HDL particle, by virtue of the antioxidative properties of its attached apo A-I, paraoxonase and glutathione peroxidase, reduces the oxidative modification of LDL by quenching the oxygen-derived free radicals generated from LDL oxidation [32]. Hence, various pharmacological interventions have been focused on raising HDL-cholesterol levels [32].

Obesity induced-IR results in profound dysregulation in the glucose homeostasis, and produces elevations in fasting and postprandial glucose levels [33]. As seen in the present study, the mean blood glucose concentration was increased in group B compared to group A. With the development of visceral obesity, the high circulating FFA leads to IR that promotes a dual effect to enhance hyperglycaemia by (i) down-regulating the insulin-sensitive glucose transporter 4 (GLUT4) via the Randle cycle and hence promotes an accumulation of glucose in the circulation and (ii) stimulating hepatic gluconeogenesis by antagonizing the action of insulin in the liver (hepatic IR) [8]. GA-treated rats in group C demonstrated a significant decrease in fasting blood glucose compared to group B. The reduction in fasting blood glucose of the GA-treated group is proposed to be accounted for by increased tissue glucose uptake via GLUT4. PPAR-γ activation in the adipose tissue has been shown to increase the expression of the c-Cbl associating protein (CAP) that is important for the translocation of GLUT4 to the cell surface [33] and inhibition of 11β-HSD1 may also exhibit similar effect by attenuating the inhibition of muscle GLUT4 translocation by active glucocorticoids [34]. These effects may therefore increase glucose disposal, giving a decrease in circulating glucose levels. More importantly, both 11β-HSD1 inhibition and PPAR-γ agonism have also been associated with reduced expression of phosphoenolpyruvate carboxykinase (PEPCK) and glucose-6-phosphatase (G6Pase) [35,36], the two rate-limiting enzymes of the gluconeogenesis pathway that is aberrantly induced in T2DM patients [34]. Uncontrolled, accelerated gluconeogenesis accounts for 90% of hepatic glucose output in T2DM patients and is thereby a significant contributor to hyperglycaemia [35].

Besides a significant reduction in blood glucose concentration in group C, mean serum insulin concentration was also reduced as compared to group B. This might be due to improved glucose-sensing proteins in the pancreatic β-cells since β-cells controls insulin secretion in response to blood glucose concentration [37]. Briefly, both the GLUT2 transporter and the enzyme glucokinase (GK) are components of the glucose-sensing apparatus of the β-cells whose expression are both decreased in diabetes. With this, the glucose threshold for insulin secretion is also decreased, leading to aberrant insulin secretion and hyperinsulinaemia. PPAR-γ activation is shown to restore both GLUT2 and GK expression [36]. Hence the glucose threshold for insulin secretion is increased, reducing insulin secretion. The HOMA-IR is used for the assessment of insulin sensitivity from basal (fasting) glucose and insulin levels; a higher value indicating lower insulin sensitivity (higher insulin resistance) and vice versa [38]. The HOMA-IR index decrease in group C was significant compared to group B - indicating an improvement in insulin sensitivity in rats on high-fat diet and given 100 mg/kg of GA.

Chronic obesity has been associated with non-adipose tissue lipid accumulation - a condition known as tissue steatosis [8,39]. During conditions of chronic caloric excess, a compensatory mechanism first occur in the leptin-responsive state whereby the surplus FFA up-regulates PPARα and promote the compensatory oxidation of the surplus FFA, with the excess energy dissipated as heat. As such process continues, such caloric excess is no longer compensated and in the leptin-unresponsive state, the surplus FFA activates PPARγ instead, leading to up-regulation of the lipogenic enzymes that cause ectopic TAG accumulation [39]. Leptin resistance has been reported to occur in late phases in both rat and human diet-induced obesity [39]. Our present study has shown that lipid depositions in all the tissues of group C rats were significantly reduced compared to group B. We postulate that such observation is accounted for by GA activation of PPARα that induces the expression of lipid-catabolizing genes such as carnitine palmitoyltransferase-1 (CPT-1), acyl CoA oxidase (ACO) and uncoupling protein (UCP-2) that are induced normally in the state of compensated caloric excess aforementioned. Tissue lipid accumulation has been associated with obesity-related IR [40,41] and these are mediated by TAG-derived metabolites that inhibits insulin signal transduction [42]. Thus, our observation of GA-mediated improvement in insulin sensitivity may be related to such decrease in tissue lipid as well.

## Conclusion

Our study has indicated that daily oral administration of 100 mg/kg of GA to high-fat dietinduced obese rats for 28 days led to significant improvement in insulin sensitivity, together with an apparent hypotriglyceridaemic and HDL-raising effect. LPL expression was upregulated mainly in the oxidative tissues and this may promote catabolism of the TAG-rich VLDL and chylomicron that usually accumulate in the circulation of MetS patients. Lastly, a decrease in tissue lipid deposition was observed with GA administration - possibly associated with an increase in fatty acid oxidation.

## Methods

### Animal treatment

The use and handling procedure of animals in this research project had been approved by the Monash University Animal Ethics Committee (AEC Approval Number: SOBSB/MY/2007/22). 24 male Sprague-Drawley rats (*Rattus norvegicus*) with an initial weight between 160 g to 200 g were randomly chosen and were housed individually in plastic cages with a 12:12-h light-dark cycle - lights starting at 0600 hours. The room was kept at 23.0°C ± 1.0°C. Before the experiment, rats in all groups were fed *ad libitum *with free access to standard rat chow (Gold Coin, Malaysia) and tap water. At the start of this experiment, each of the 3 groups were subjected to different dietary composition and treatment - Group A was fed on a normal diet; Group B was fed on high-fat diet; Group C was fed on high-fat diet and given 100 mg/kg of GA through a feeding bottle. The high-fat diet pellets were prepared by mixing powdered rat chow to vegetable shortening in a ratio of 2:3. All three groups of rats were fed and treated for 28 days. The amount of food and water or GA consumed was recorded daily.

### Sample collection

Rats in all groups were fasted for approximately 12 hours prior to sacrifice. The rats were anaesthetized by intraperitoneal injection of 150 mg/kg of sodium pentobarbital (Nembutal) and dissection was carried out between 0800 and 1000 hours. Blood was drawn from the apex of the cardiac ventricle and five drops of blood were collected into a microcentrifuge tube containing a mixture of EDTA and NaF in a 1:2 (w/w) ratio. This aliquot of blood will be used for blood glucose determination. The remaining blood sample was collected into a sterile Falcon tube and centrifuged at 12,000 × g for 10 minutes. The serum supernatant was aliquoted into microcentrifuge tubes and stored at -80°C until required for analysis. The seven tissues of interest, namely, the heart, kidney, liver, AM, QF, VAT and SAT were promptly harvested and immediately flash-frozen in liquid nitrogen and stored at -80°C.

### Blood biochemistry analysis

Blood glucose was determined using the Trinder's glucose oxidase method while serum insulin was determined using the Rat/Mouse Insulin ELISA Kit (Linco Research, USA). The HOMA-IR was calculated as the product of fasting blood glucose and serum insulin divided by 22.5 [38]. Serum FFA, TAG and total cholesterol were determined using the Zenbio 96- well Serum/Plasma Fatty Acid Kit Non-Esterified Fatty Acids Detection 100 point kit (Zenbio, USA), Randox Triglycerides Kit (Randox, UK) and Randox CH200 Cholesterol Kit (Randox, UK) respectively. HDL-cholesterol was determined using the aforementioned cholesterol kit after precipitation of HDL using the Randox CH203 HDL Precipitant Kit (Randox, UK). LDL-cholesterol was calculated using the levels of total cholesterol, TAG, HDL-cholesterol obtained using the Friedewald formula [43].

### Real time reverse transcription polymerase chain reaction (qRT-PCR) of LPL gene

Qiagen RNeasy Mini kit (Qiagen, USA) was used to isolate the total RNA from the liver, kidney, AM and QF while Qiagen RNeasy Lipid Tissue Mini kit was used to isolate the total RNA from SAT and VAT (Qiagen, USA). The purity of the RNA was determined by determining the absorbance values at 260 nm and 280 nm (A_260_/A_280 _ratio) where a ratio of 1.9-2.1 is considered pure. Promega RQ1 RNase-free DNase (Promega, USA) was used for the RNase-free DNase treatment and lastly, Qiagen Omniscript Reverse Transcriptase kit (Qiagen, USA) was used for cDNA synthesis. Relative LPL expression was determined by qT-PCR using the Comparative Ct (ΔΔCt) Method, normalized to the BAC gene. Sequence of primers and probes specific to *Rattus norvegicus *LPL [GenBank: BC081836] and BAC [GenBank: BC063166] mRNA are as follows:

LPL, forward: 5'-CAGCAAGGCATACAGGTG-3'

LPL, reverse: '-CGAGTCTTCAGGTACATCTTAC-3'

LPL, probe: 5'-(6-FAM) TTCTCTTGGCTCTGACC(BHQ1)-3'

BAC, forward: 5'-GTATGGGTCAGAAGGACTCC-3'

BAC, reverse: 5'-GTTCAATGGGGTACTTCAGG-3'

BAC, probe: 5'-(TET) CCTCTCTTGCTCTGGGC(BHQ1)-3'

Agarose gel electrophoresis was performed on amplicons produced from qRT-PCR to assess primer specificity.

### Tissue lipid quantification

Frozen tissues were cut into cubes of 5 × 5 × 5 mm on a sterile petri dish and fixed onto a cryotome at -25°C using the Optimal Cutting Temperature (OCT) Compound (Leica, Germany). Sections of 5 μm thick were stained with ORO using the method of Koopman *et al. *[44] and captured at 400× magnification. Lipid depositions for all tissues were calculated as per Goodpaster *et al. *[40]. Briefly, captured images were converted to gray scale on Image J software and the threshold for staining intensity was adjusted to selectively pick up lipid droplets. Pixels with intensities of 150 ± 30 arbitrary unit (AU) were selected (intensity ranges from 0AU to 255AU; 0AU represented complete staining and 255AU represented no staining). The mean of eight values from eight contiguous views captured was used to determine the level of lipid deposition in arbitrary units (AU).

### Statistical analysis

Statistical analysis for LPL expression was performed using the Relative Expression Software Tool (REST^©^) MCS Beta 2006. All other data were analyzed using Statistics Package for the Social Sciences (SPSS) for Windows Version 16.0. Data distribution was analyzed using the Kolmogorov-Smirnov normality test. Data with parametric distribution were then analyzed using Analysis of Variance (ANOVA) and if the results were significant, a Post Hoc Scheffe test was performed. Data with non-parametric distribution was analyzed using Kruskall-Wallis test. All data in this study were found to be parametric and hence were all reported as mean ± standard error. In all analyses, a p-value of ≤ 0.05 was considered statistically significant.

## Abbreviations

11β-HSD: 11β-hydroxysteroid dehydrogenase; ACTH: adrenocorticotrophic hormone; ACO: acyl CoA oxidase; AM: abdominal muscle; apo: apolipoprotein; BAC: β-actin; CAP: c-Cbl associating protein; CPT-1: carnitine palmitoyltransferase-1; FFA: free fatty acids; GA: glycyrrhizic acid; GE: glycyrrhetic acid; GK: glucokinase; GLUT: glucose transporter; HDL: high-density lipoprotein; HOMA-IR: homeostasis model assessment of insulin resistance; IR: insulin resistance; LDL: low-density lipoprotein; LPL: lipoprotein lipase; MetS: metabolic syndrome; ORO: Oil Red O; PEPCK: phosphoenolpyruvate carboxykinase; G6Pase: glucose- 6-phosphatase; PPAR: peroxisome proliferator-activator receptor; PPRE: peroxisome proliferator response element; QF: quadriceps femoris; SAT: subcutaneous adipose tissue; T2DM: type 2 diabetes mellitus; TAG: triacylglycerol; TNF-α: tumour necrosis factor-alpha; TZDs: thiazolidinediones; UCP-2: uncoupling protein; VAT: visceral adipose tissue; VLDL: very-low-density lipoprotein; WHHL: watanabe heritable hyperlipidemic.

## Competing interests

The authors declare that they have no competing interests.

## Authors' contributions

CHAE performed all data acquisition, analysis and interpretation and manuscript preparation. WYAL was involved in data interpretation and manuscript preparation. TSH and KAK participated in the coordination of the study and helped in drafting the manuscript. All authors read and approved the final manuscript.
